# Oxford Textbook of Palliative Medicine 2E

**DOI:** 10.1054/bjoc.1999.1134

**Published:** 2000-03-21

**Authors:** A Tookman

**Affiliations:** Edenhall Marie Curie Centre, Consultant Palliative Medicine, Royal Free Hospital Trust, London, UK


*British Journal of Cancer* (2000) **82**(8), 1492
© 2000 Cancer Research Campaign
DOI: 10.1054/ bjoc.1999.1134, available online at http://www.idealibrary.com on IDEAL®

### Book Review

### Oxford Textbook of Palliative Medicine 2E

The second edition of the *Oxford Textbook of Palliative Medicine* is a very good book, better than the first edition, being far more organized and consistent in its style. There is a wealth of information in its 1300 pages demonstrating the diverse nature of the skills needed in palliative care, however, some of the chapters need a degree of expertise to interpret them in the correct context. This is not the book for those who want a 'manual on palliative care' or a book on 'how to symptom control'. There are many excellent small volumes on symptom control in patients with advanced cancer. This text book is clearly a reference book.

The *Oxford Textbook of Palliative Medicine* illustrates the distinctive skills that are needed by the palliative medicine physician. There are a wide range of issues covered in its 22 sections, and this is to be expected in what is undoubtedly the major textbook of palliative care. The largest section is (as one would expect) is on symptom management with approximately 37 chapters. I found some chapters in this section frustrating since they were a little pedestrian in their approach. Other chapters were stimulating but let down by misinformation. A good example of this is the chapter on the palliation of respiratory symptoms. This is an excellent chapter which reflects the expertise that the author has in this area, however, I suspect many oncologists would criticize the comments made about the place and use of chemotherapy and radiotherapy in patients with lung cancer. In addition I found that the discussion about the psycho-social management of breathlessness was limited, which is surprising considering the significant body of literature that supports this approach.

The chapters on disease modifying management are excellent and more up-to-date than some chapters. These strong chapters on clinical and medical oncology are welcomed – they will undoubtedly be very useful to the palliative medicine physician, emphasizing the need for there to be a close collaborative working relationship between them and the oncologist.

One is always selective when reading a large textbook and one cannot help but be drawn to chapters in which there is a personal interest. The section on rehabilitation is, for me, one of those sections. In my opinion this is an area of cancer care that is undervalued and under-recognized by both oncologists and palliative medicine physicians. Although the section contained some useful information, it was limited and gave no sense of the value of teamwork that is so vital for effective rehabilitation in palliative care patients.

Should this textbook be on oncologists' bookshelves? Perhaps it should. However, it would be far more appropriate for oncologists to continue to develop a close working relationship with their local palliative care team (who certainly should have this textbook on their bookshelf). When faced with difficult symptoms or complex palliative care issues access to the *Oxford Textbook of Palliative Medicine* would be helpful to empower this team to make quality decisions about management.

Patients with cancer are often described as being on a journey. To truly understand the multi-dimensional needs of the patient, one must recognize the point they have reached on their individual cancer journey. To truly understand the *Oxford Textbook of Palliative Medicine* one must appreciate that it is on a journey too. It is clearly getting better but it still has some way to go. The cross-referencing needs to be improved and the important contribution of the specialist nurse needs to be more explicit. There needs to a greater emphasis on the role of specialist palliative care in non-cancer conditions. However, it is easy to criticize and I have enormous admiration for the considerable effort the authors have made in collecting together the contents of this textbook. It is unique. It is the reference book used by all palliative medicine physicians across the world and there is no competitor. It undoubtedly is a volume that highlights the specialist skills of the palliative medicine physician and reinforces that palliative medicine, as a specialty, has truly 'come of age'.

*Dr A Tookman*
*Medical Director,*
*Edenhall Marie Curie Centre,*
*Consultant Palliative Medicine,*
*Royal Free Hospital Trust,*
*London, UK*

